# Type I gamma phosphatidylinositol phosphate kinase modulates invasion and proliferation and its expression correlates with poor prognosis in breast cancer

**DOI:** 10.1186/bcr2471

**Published:** 2010-01-14

**Authors:** Yue Sun, Dmitry A Turbin, Kun Ling, Narendra Thapa, Samuel Leung, David G Huntsman, Richard A Anderson

**Affiliations:** 1 Department of Pharmacology, University of Wisconsin Medical School, 1300 University Avenue, Madison, Wisconsin, 53706, USA; 2 Genetic Pathology Evaluation Centre of the Prostate Centre, University of British Columbia, Room 509, JBRC, 2660 Oak St., Vancouver, BC, V6H 3Z6, Canada

## Abstract

**Introduction:**

The loss of E-cadherin based cell-cell contacts and tumor cell migration to the vasculature and lymphatic system are hallmarks of metastasis of epithelial cancers. Type I gamma phosphatidylinositol phosphate kinase (PIPKIγ), an enzyme that generates phosphatidylinositol 4,5-bisphosphate (PI4,5P_2_) a lipid messenger and precursor to many additional second messengers, was found to regulate E-cadherin cell-cell contacts and growth factor-stimulated directional cell migration, indicating that PIPKIγ regulates key steps in metastasis. Here, we assess the expression of PIPKIγ in breast cancers and have shown that expression correlated with disease progression and outcome.

**Methods:**

Using a tissue microarray, we analyzed 438 breast carcinomas for the levels of PIPKIγ and investigated the correlation of PIPKIγ expression with patient survival via Kaplan-Meier survival analysis. Moreover, via knockdown of the expression of PIPKIγ in cultured breast cancer cells with siRNA, the roles of PIPKIγ in breast cancer migration, invasion, and proliferation were examined.

**Results:**

Tissue microarray data shows that ~18% of the cohort immunostained showed high expression of PIPKIγ. The Kaplan-Meier survival analysis revealed a significant inverse correlation between strong PIPKIγ expression and overall patient survival. Expression of PIPKIγ correlated positively with epidermal growth factor receptor (EGFR) expression, which regulates breast cancer progression and metastasis. In cultured breast cancer cells, PIPKIγ is required for growth factor stimulated migration, invasion, and proliferation of cells.

**Conclusions:**

The results reveal a significant correlation between PIPKIγ expression and the progression of breast cancer. This is consistent with PIPKIγ 's role in breast cancer cell migration, invasion, and proliferation.

## Introduction

Breast cancer is one of the most significant malignancies of women [1,2]. Despite successful treatment of the primary malignancy, tumors relapse and subsequent metastasis often occur at distant sites, including bone, lung, liver, and brain [3-5]. The presence of breast cancer metastasis significantly influences patients' prognosis. The five-year survival rate from breast cancer drops from 96% to 75% with regional spread, and drops to 20% with distant spread [6,7]. Understanding the molecular mechanism that regulates breast cancer metastasis is critical to developing therapies to treat breast cancer and increase the survival rate; and defining markers that predict metastatic potential will be a key for defining therapeutic approaches.

Phosphatidylinositol 4,5-bisphosphate (PI4,5P_2_) plays central roles in regulating cell migration, a key step of cancer metastasis, by modulating adhesion turnover and dynamic cytoskeleton rearrangements [8]. By interacting with cofilin, PI4,5P_2 _regulates the elongation of newly polymerized actin filaments via controlling cofilin cellular distribution and activation [9,10]. Including cofilin, PI4,5P_2 _also regulates the reorganization of actin cytoskeleton by associating with other proteins such as α-actinin, WASP/N-WASP, gelsolin, profilin, and villin [8,11]. Furthermore, PI4,5P_2 _regulates adhesion turnover by binding to and modulating talin, vinculin, ezrin/radixin/moesin, calpain, and other proteins involved in adhesion dynamics [8,11].

Type I gamma phosphatidylinositol phosphate kinase (PIPKIγ) is one of the major enzymes in cells that generate PI4,5P_2 _by phosphorylation of phosphatidylinositol(4)phosphate [12]. Via the spatial and temporal control of PI4,5P_2 _synthesis_, _PIPKIγ plays a key role in multiple biological processes [8,13-18]. Loss of PIPKIγ leads to defects of cardiovascular and neuronal development, which is consistent with changes in cadherin function and cell migration [19]. PIPKIγ is also required for the integrity of the membrane cytoskeleton [20]. The PIPKIγ is alternatively spliced in cells, resulting in at least two major variants, PIPKIγ635 and PIPKIγ661 (now named PIPKIγi1 and PIPKIγi2), which differ by a 26 amino acid C-terminal extension [21,22]. The short splicing variant PIPKIγi1 is reported to be a major contributor of the PI4,5P_2 _pool that supports G protein-coupled receptor-mediated inositol 1,4,5-trisphosphate generation and plays a critical role in Ca^2+ ^flux [23]. Unlike PIPKIγi1, PIPKIγi2 binds to talin via the 26 amino acid C-terminal extension in a process regulated by tyrosine phosphorylation, targeting PIPKIγi2 to adhesions [17,24]. The phosphorylation is mediated by both growth factor receptors and by the non-receptor tyrosine kinase, Src [8,17,18]. By generating PI4,5P_2 _and regulating talin assembly, PIPKIγi2 modulates nascent adhesion formation at the leading edge to facilitate cell migration. Specifically, the PIPKIγi2 regulates epidermal and other growth factor stimulated chemotaxis [25], a process key to intravasation of cancer cells where cells migrate into the vasculature and lymphatic system, a first step in the metastasis of breast cancers [9].

The loss of E-cadherin cell-cell contacts is a hallmark for the progression of cancers of epithelial origin [26]. Remarkably, the PIPKIγ also regulates the ability of epithelial cells to assemble E-cadherin-based cell-cell contacts [16]. This occurs by an association of PIPKIγi2 with E-cadherin and the recruitment of specific clathrin adaptors required for basolateral and endocytic trafficking by an association with the PIPKIγi2 C-terminus. A loss of PIPKIγ expression leads to a loss of E-cadherin targeting to the plasma membrane and a loss of E-cadherin-based cell-cell contacts [16]. Thus, PIPKIγ regulates the plasma membrane targeting of E-cadherin-based cell-cell contacts and the polarization of epithelial cells. The positioning of the PIPKIγ as a regulator of both E-cadherin cell-cell contact assembly and growth factor stimulated cell migration positions the PIPKIγ as a key-signaling molecule in physiological functions that are fundamental to the metastasis of cancers of epithelial origin.

In spite of the increasing evidence indicating that PIPKIγ plays a crucial role in cell migration so that it is likely implicated in cancer metastasis, the pathological correlation between the lipid kinase and cancer progression remains uninvestigated. Here, we analyzed breast carcinomas via tissue microarray analyses for the levels of PIPKIγ and demonstrated a significant inverse correlation between strong positive PIPKIγ expression and overall survival. In addition, the requirement of PIPKIγ for the migration, invasion, and growth of breast cancer cells has been confirmed using *in vitro *models.

## Materials and methods

### Antibody

Polyclonal PIPKIγ anti-serum was generated from rabbit (Covance, Princeton, NJ, USA) using purified His-tagged PIPKIγ. Anti-serum was purified on an affinity column generated by coupling recombinant C-terminus of PIPKIγ to cyanogen bromide-activated Sepharose 4B (Sigma-Aldrich, Saint Louis, MO, USA) as described [17,25,27]. The affinity-purified antibody recognizes both PIPKIγi1 and PIPKIγi2.

### Constructs

The siRNA sequence targeting PIPKIγ is 5'-GGACCUGGACUUCAUGCAG-3'. The sequence of control scrambled siRNA is 5'-GUACCUGUACUUCAUGCAG-3'. Oligonucleotide sequences used for generation of short hairpin RNA (shRNA) specific for PIPKIγ were: GCCACCTTCTTTCGAAGAA (PIPKIγ shRNA) and GCCTTCTTCGCTAAACGAA (Control shRNA). Generation of replication-defective infectious viral particles and the transduction of the cells were carried out following the protocol provided by Addgene (Addgene Inc., Cambridge, MA, USA). In brief, synthesized oligonucleotides were annealed and cloned into HpaI and XhoI sites of pLL3.7 vector (Addgene Inc., Cambridge, MA, USA). Stabl3 competent cells (Invitrogen, Carlsbad, CA, USA) were used for transformation and DNA purification to minimize the mutagenesis. The integrity of lentiviral vector-containing cloned shRNA sequences were validated by DNA sequencing.

### Cell cultures and transfection

MDA-MB-231 and MDA-MB-435S cells were cultured using DMEM supplemented with 10% FBS. SKBR3 cells were cultured in DMEM/F12 with 10% FBS. For siRNA transfection, cells were transfected with Oligofectamine (Invitrogen, Carlsbad, CA, USA) following the manufacturer's instructions.

### Patient information and tissue microarrays

A tissue microarray was constructed out of 438 archival invasive breast carcinoma samples at Vancouver General Hospital. These cases were collected between 1974 and 1995. Ethics board approval was obtained for all cases. Patients' demographics, pathological features of the tumors and expression of various biomarkers have been reported previously [28]. In brief, median age of the patients at the moment of diagnosis was 61.5 years; median survival time was 11.9 years. Histological distribution included 379 infiltrating ductal carcinoma, 41 infiltrating lobular carcinoma and 8 special types.

Three tissue microarray blocks were assembled using a manual tissue microarrayer (Beecher Instruments, Inc., Silver Springs, MD, USA) from formalin fixed paraffin-embedded tissue as described previously [29]. Tissue sections (4 μm thick) were cut from the donor blocks and stained with hematoxylin and eosin for tissue review. Representative areas of tumor were circled on the slides and corresponding donor blocks; duplicate 0.6 mm cores were taken from these blocks and inserted into three recipient blocks. Sections (4 μm thick) were cut from the recipient blocks and deparaffinized with CitriSolve and dehydrated through three alcohol changes. Antigen retrieval was performed using a steamer for 30 minutes in 0.1 M citrate buffer (pH 6.0). After that, sections were rinsed with PBS three times for five minutes each time. Hydrogen peroxide and serum free protein block (Dako, Carpenteria, CA, USA) were used to block endogenous peroxidases and prevent non-specific protein binding. Sections were then incubated with anti-PIPKIγ antibody (0.5 μg/ml) in a sealed immunochamber overnight at 4°C and Dako Envision anti-rabbit secondary antibody was applied at room temperature for 30 minutes. The NovaRed Substrate Kit (Vector Labs, Burlingame, CA, USA) was used to visualize the protein. Slides were then counterstained with hematoxylin and mounted.

### Digital image database

The hematoxylin and eosin and immunohistochemistry images of all cores used in this study are publicly available at the companion site [30]. The site was constructed using a GPEC database and a Java applet provided by Bacus Laboratories, Inc (Bacus Laboratories, Inc., Lombard, IL, USA). All the slides were scanned with a BLISS scanner (Bacus Laboratories, Inc., Lombard, IL, USA), and posted on the site. WebSlide Browser for Windows (Bacus Laboratories, Inc., Lombard, IL, USA) can be used for viewing preview images of the arrays and images of individual cores.

### Cell migration assay

The assays were performed in modified Boyden chamber transwell (Neuroprobe, Gaithersburg, MD, USA) as described [31,32]. The membrane was pre-coated with type I collagen (10 μg/ml). Per well, 50,000 cells were applied. Chemotaxis assays were performed at 37°C in humidified air with 5% CO_2 _for four hours. Cells migrated through to the underside of the membrane were counted in five high power fields, in a blinded fashion. The migration index for each experiment was calculated as the mean number of cells that migrated toward medium-containing 1% FBS divided by the mean number of cells that migrated toward medium-containing BSA only.

### Invasion assay

Matrigel-coated Transwells (BD Bioscience, San Jose, CA, USA) were incubated with DMEM for four hours and 5 × 10^4 ^cells were plated in the upper chambers. The lower chambers contained 1% FBS conditioned DMEM. The inserts were incubated at 37°C in humidified air with 5% CO_2 _for 24 hours. The cells that had invaded the lower surface of the membrane were fixed with 4% polyformaldehyde and stained with 0.2% crystal violet. The number of cells that had invaded was quantified by counting random fields using a light microscope.

### Cell proliferation assay

MDA-MB-231, MDA-MB-435S, or SKBR3 cells infected with lentivirus containing either control shRNA or PIPKIγ shRNA were seeded into 12-well culture plates at a density of 1000 cells/well. Then, manual cell counting was performed every two days for eight days.

### Statistical analysis

We performed Kaplan-Meier survival analysis using log-rank test to determine differences in survival of the patients with different levels of PIPKIγ protein expression. Breslow test was also used to emphasize survival differences in the first 5 to 10 years of the follow up. The alpha level was determined as 5%. All the tests were two-sided. Spearman's correlation was utilized to estimate correlations between PIPKIγ protein content with that of other biomarkers and clinical prognostic factors.

## Results

### Expression of PIPKIγ in breast carcinomas

The cellular functions of PIPKIγ suggest the potential for roles in epithelial cancer progression. To explore the potential that PIPKIγ may correlate with disease progression and outcome we began to assess the content of PIPKIγ in a well-characterized tissue microarray of breast cancer patients. For this approach we used well-characterized antibody that specifically detect all splice variants of the PIPKIγ as described in Materials and Methods. As a control, PIPKIγ expression was knocked down with siRNA (Small interfering RNA) in breast epithelial (MCF10a) or carcinomas cell lines and this resulted in a loss of protein by western blotting and a loss of the immunostaining in these cells [16,25].

**Figure 1:**
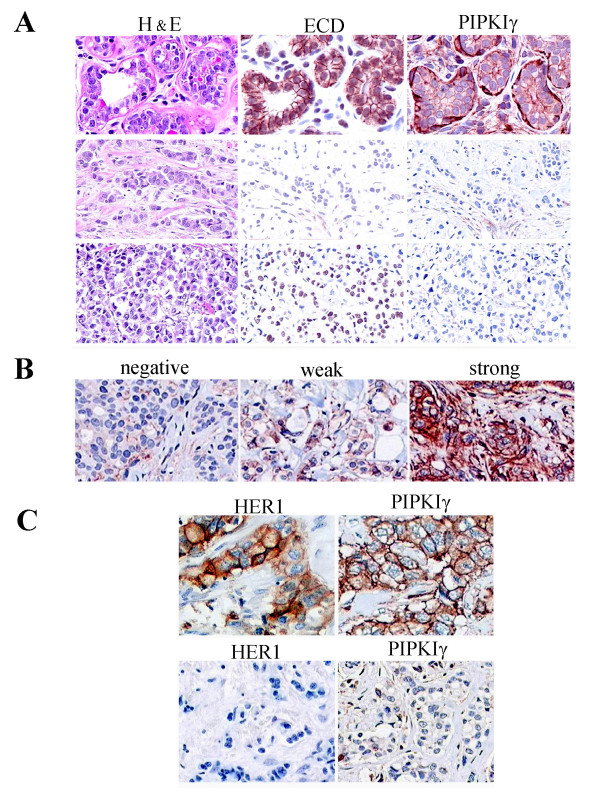
**Expression of PIPKIγ in breast carcinomas**. **(a) **Shown is H&E, E-cadherin and type I gamma phosphatidylinositol phosphate kinase (PIPKIγ) staining of normal breast tissue (top). Breast carcinomas that show a loss of both E-cadherin and membrane PIPKIγ (middle). A fraction of carcinomas that show both a mistargeting of E-cadherin and a loss of PIPKIγ (bottom). **(b) **Expression of PIPKIγ in breast carcinomas. (Left panel) Representative breast carcinomas stained negative for PIPKIγ. (Middle panel) Representative breast carcinomas stained weakly positive for PIPKIγ. (Right panel) Representative breast carcinomas stained strong positive for PIPKIγ. **(c) **Immunohistochemical staining of breast tumor tissue for human epidermal growth factor receptor (HER-1) and PIPKIγ. The panels on the top or on the bottom are from the same tumors. The top panels show strong expression of HER-1 and PIPKIγ, whereas the bottom shows weak staining for both antigens.

Normal breast tissues show strong staining for E-cadherin epithelia cells lining the ducts and PIPKIγ also shows strong staining of these cells (Figure 1a). To assess how the PIPKIγ expression changes within breast cancers we have screened a breast cancer tissue microarray. The expression of PIPKIγ in breast carcinomas was demonstrated via tissue microarray that was constructed out of 438 archival invasive breast carcinoma samples at the Vancouver General Hospital [33]. Of the 438 breast carcinoma represented on the tissue microarray, 330 specimens (75.3%) were considered interpretable. As shown in the lower panels of Figure 1a, in tumors with a loss of E-cadherin there is also a loss of PIPKIγ staining. The bottom panel of Figure 1a shows that in a fraction of tumors E-cadherin was expressed but not targeted to the plasma membrane and these tumors also lost PIPKIγ. These combined data are shown statistically in Table 1.

**Table 1:** Correlation of expression of PIPKIγ protein with different biomarkers

Biomarker	PIPKIγ negative and weak vs. strong staining		
	
	Spearman's correlation coefficient	*P *value	Number of cases
E-cadherin negative vs. weak and strong staining	0.106	0.046	351

ER at 1% cut-off point	-0.256	0.000003	327

PR at 1% cut-off point	-0.298	0.000001	256

EGFR (HER1) negative vs. weak and strong staining	0.262	0.000003	306

HER2/neu	0.171	0.003	309

Nottingham Grade	0.185	0.001	346

Tumour size	0.025	0.648	349

Nodal status	0.012	0.828	314

EGFR = epidermal growth factor receptor; ER = estrogen receptor; HER2 = human epidermal growth factor receptor 2; PIPKIγ = type I gamma phosphatidylinositol phosphate kinase; PR = progesterone receptor.

In Figure 1b, examples of the different PIPKIγ staining of breast tumors are shown. The slides were scored as 'negative' if no PIPKIγ staining was detected, 'weak staining' if there was any amount of weak membranous staining and/or strong membranous staining in less than 50% of tumor cells, and 'strong staining' if there was strong membranous staining in 50% or more carcinoma cells. Among the 358 specimens analyzed, 149 specimens were PIPKIγ staining negative (41.6%), 144 specimens were PIPKIγ staining weakly positive (40.2%), and 65 specimens were PIPKIγ staining strongly positive (18.2%) (Table 2).

**Table 2:** Number of breast carcinomas cases stained with negative, weakly positive and strong positive PIPKIγ

PIPKIγ staining	Cases	% of total cases
negative	149	41.6
weakly positive	144	40.2
strong positive	65	18.2

PIPKIγ = type I gamma phosphatidylinositol phosphate kinase.

### Survival analysis

**Figure 2:**
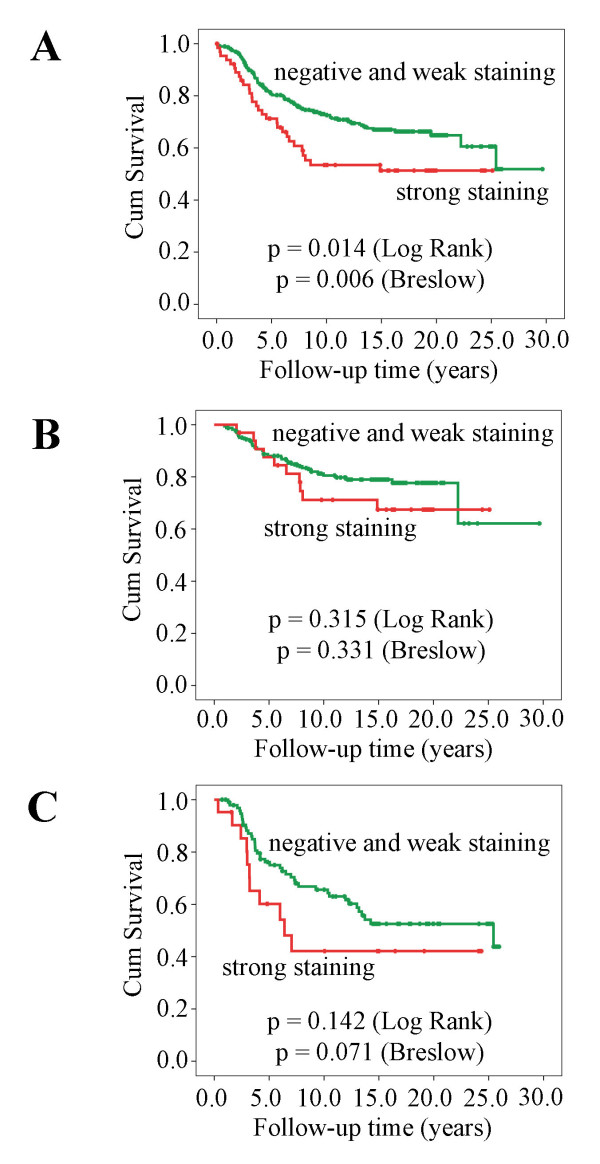
**Kaplan-Meier survival curves for PIPKIγ staining, with time given in years**. **(a) **The entire cohort of patients. **(b) **Lymph node negative patients. **(c) **Lymph node positive patients. PIPKIγ = type I gamma phosphatidylinositol phosphate kinase.

To investigate if PIPKIγ tissue content correlates to breast cancer prognosis, Kaplan-Meier survival curves were generated using PIPKIγ antibody staining. As shown in Figure 2a, these curves show that strong positive PIPKIγ expression was correlated to poor outcome (Log Rank chi-squared = 6.078, *P *= 0.014; Breslow chi-squared = 7.454, *P *= 0.006). It indicates that PIPKIγ expression is inversely correlated to the survival of breast cancer patients. To further define the correlation of PIPKIγ expression with breast cancer prognosis, patients were stratified based on the lymph node status. Kaplan-Meier survival curves were generated for lymph node negative and lymph node positive. As shown in Figure 2b, there was no significant difference in survival of the patients with PIPKIγ strong positive and weak positive or negative tumor in lymph node negative subset (Log Rank chi-squared = 1.01, *P *= 0.315; Breslow chi-squared = 0.945, *P *= 0.331). In the lymph node positive subset, the longer term survival difference was not significant in the log-rank test (Log Rank chi-squared = 2.154, *P *= 0.142, Figure 2c); however, the 5- and 10-year survival rate of PIPKIγ strong positive patients is significantly lower than PIPKIγ negative or weakly positive patients and had trend towards significance in Breslow test (Breslow chi-squared = 3.267, *P *= 0.071, Figure 2c). Considering that the median age of the patients at the moment of diagnosis is 61.5 years, the longer-term survival as 20 or 25 years may not clearly reflect the survival of breast cancer because it is very close to the normal average human life limit. The 5- and 10-year survival rate difference supports that PIPKIγ expression is inversely correlated to the survival of breast cancer patients.

### Correlation of PIPKIγ expression with breast cancer biomarkers

To further characterize PIPKIγ expression in breast cancer, Spearman's correlation was used to demonstrate the correlation of PIPKIγ expression with clinical characteristics such as lymph nodal status, tumor size and Nottingham tumor grade. As shown in Table 1, PIPKIγ expression does not correlate significantly with lymph nodal status and tumor size. There is a weak significant correlation with the Nottingham grade.

PIPKIγ expression was correlated with the expression of some established breast cancer biomarkers on a tissue microarray. From this analysis there was a correlation between the loss of PIPKIγ with loss of E-cadherin expression and plasma membrane targeting. It is consistent with the finding that PIPKIγ directly binds to E-cadherin and modulates E-cadherin trafficking [16]. Epidermal growth factor receptor (EGFR)/human epidermal growth factor receptor (HER) family receptors are often overexpressed in breast cancers and are used as breast cancer biomarkers [34]. Interestingly, our results show that both HER1 (EGFR) and HER2/neu expression are correlated with PIPKIγ expression (Table 1). EGFR/HER family receptors play key roles in breast cancer metastasis and their expression are correlated with worse prognostic patient survival rates [35,36]. The correlation of PIPKIγ with EGFR/HER family receptors is consistent with the finding that PIPKIγ expression is inversely correlated to the survival of breast cancer patients. In addition, the results of tissue microarrays show that the expression of PIPKIγ is inversely correlated to estrogen receptor (ER) and progestin receptor (PR). ER and PR status are important for distinguishing different breast cancer subtypes and they are critical for ER^+ ^or PR^+ ^breast cancer cell growth [37,38]. Our finding is the first time to discover a correlation between PIPKIγ with ovarian hormone receptors ER and PR.

### PIPKIγ regulates growth factor stimulated breast cancer migration and invasion

**Figure 3:**
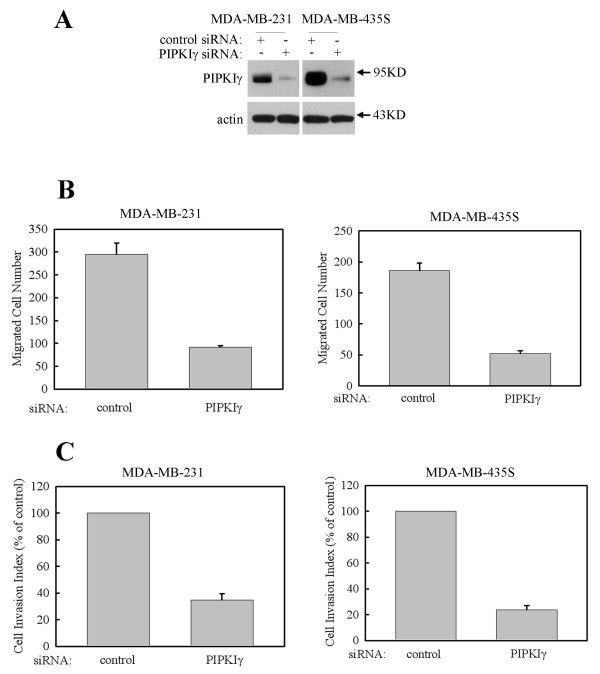
**Knockdown of PIPKIγ blocks breast cancer cell line migration and invasion**. MDA-MB-231 or MDA-MB-435S cells were transfected with control siRNA, or type I gamma phosphatidylinositol phosphate kinase (PIPKIγ) specific siRNA separately as indicated. **(a) **Expression of PIPKIγ and actin were detected by their specific antibodies. **(b) **FBS (1%) induced migration of MDA-MB-231 and MDA-MB-435S cells. **(c) **FBS (1%) induced invasion of MDA-MB-231 and MDA-MB-435S cells.

Previously, it has been shown that PIPKIγ is required for growth factor-induced HeLa (Helen Lane) cell migration [25]. As PIPKIγ expression is inversely correlated to the survival of breast cancer patients, to regulate cancer cell metastasis might be one of the mechanisms for PIPKIγ function in breast cancer. In order to verify this possibility, the effect of PIPKIγ knockdown on two breast cancer cell lines, MDA-MB-231 and MDA-MB-435S, migration and invasion was demonstrated. As shown in Figure 3a, PIPKIγ-specific siRNA could specifically knockdown expression of PIPKIγ in MDA-MB-231 or MDA-MB-435S cells, compared with the actin control. The effect of PIPKIγ-knockdown on breast cancer cell migration was quantified using a modified Boyden chamber transwell assay. As shown in Figure 3b, the knockdown of PIPKIγ blocked migration of both MDA-MB-231 and MDA-MB-435S. To investigate the effect of PIPKIγ-knockdown on breast cancer cell invasion, a Matrigel invasion assay was used. As shown in Figure 3c, the knockdown of PIPKIγ also blocked invasion of both MDA-MB-231 and MDA-MB-435S.

### PIPKIγ regulates breast cancer cell growth

**Figure 4:**
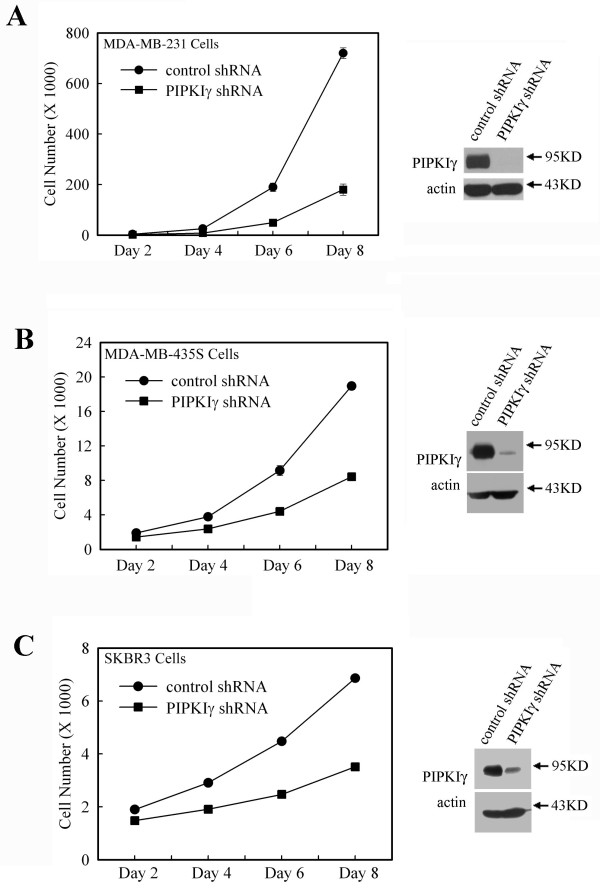
**PIPKIγ expression is required for rapid cell proliferation in breast cancer cells**. Lentiviral vector was used to generate the stable knockdown of type I gamma phosphatidylinositol phosphate kinase (PIPKIγ) in breast cancer cell lines as described in "Material and methods". Decreased expression of PIPKIγ was assessed by using PIPKIγ specific antibody. Cells infected with either Control short-hairpin RNA (shRNA) or PIPKIγ shRNA were seeded into 12-well culture plates at a density of 1000 cells/well. Manual cell counting was performed every two days for the eight days. The cell numbers were counted from at least three wells for each cell type and expressed as mean ± standard deviation from one representative experiment. **(a) **MDA-MB-231 cells. **(b) **MDA-MB-435S cells. **(c) **SKBR3 cells.

To further investigate the role of PIPKIγ in the pathogenesis of breast cancer progression, lenti-virus-vector-based PIPKIγ shRNA was used to establish PIPKIγ-knockdown stable cell lines in MDA-MB-231, MDA-MB-435S, and SKBR3 cells. The knockdown of PIPKIγ expression in these stable cell lines was confirmed by western blot as shown in Figure 4. The effect of PIPKIγ-knockdown on breast cancer cell growth was then determined. As shown in Figure 4, PIPKIγ-knockdown decreased cell growth of MDA-MB-231, MDA-MB-435S, and SKBR3 cells (Figures 4a to c). This result indicates an important role of PIPKIγ in breast cancer cell proliferation and is consistent with the finding that PIPKIγ expression is inversely correlated to the survival of breast cancer patients.

## Discussion

Here, we have shown that the expression of PIPKIγ is inversely correlated to the survival of breast cancer patients, indicating a potential prognostic value of PIPKIγ. Consistently, PIPKIγ is required for breast cancer cell migration, invasion, and proliferation. All these results support the fact that PIPKIγ plays an important role in breast cancer progression.

Overexpression of the EGFR has been shown to correlate with metastasis and poor prognosis of breast cancer [39]. Our results show that EGFR expression is correlated to PIPKIγ expression in breast cancer cells, which hints that EGFR and PIPKIγ may cooperate to facilitate breast cancer metastasis. Upon EGF stimulation, EGF-induced phosphorylation of PIPKIγ causes a disassembly of the phospholipase C-γ1-PIPKIγ complex and this could enhance PI4,5P_2 _accumulation and thus enhances talin assembly into adhesions and this in turn would facilitate the protrusion formation and stabilization of adhesions, which is required for cell migration [25].

Our results demonstrate that PIPKIγ is not only required for breast cancer cell migration but also for breast cancer cell invasion. During invasion, cancer cells form actin-containing protrusions, called invadopodia, that extend into the extracellular matrix and participate in extracellular matrix degradation [40]. ADP-ribosylation factor 6 (ARF6) is a regulator of invadopodia formation and cell invasion [41] and ARF6 directly activates PIPKIγ [42]. It is plausible that PIPKIγ plays a role in invadopodia formation by production of PI4,5P_2 _that regulates actin filament dynamics via cofilin, α-actinin, and vinculin.

The PIPKIγ expression is correlated with E-cadherin in our tissue microarray results. It is consistent with the role of PIPKIγ in regulating E-cadherin trafficking. PIPKIγ binds directly to E-cadherin and recruits clathrin adaptor complexes AP1B to the E-cadherin-PIPKIγ complex and this controls the targeting of E-cadherin to the basolateral membrane [14,16]. The loss of PIPKIγ results in the loss of E-cadherin targeting to the plasma membrane and a loss of epithelial cell polarization. The PIPKIγ-interacting region in E-cadherin has some overlapping with β-catenin-binding domain [16]. PIPKIγ may regulate E-cadherin-β-catenin interaction and then modulate β-catenin nuclear translocation. Transactivation of β-catenin correlated significantly with cyclin D1 expression, and that high β-catenin activity significantly correlated with poor prognosis of the patients and was a strong and independent prognostic factor in breast cancer [43]. PIPKIγ may regulate breast cancer progression via E-cadherin-β-catenin signal pathway.

Breast cancers are heterogeneous in their ER and PR status and display different response to tamoxifen treatment [44]. A study on the cellular phenotypes of breast cancer tumors in 19,541 white women with node-negative disease showed that ER+/PR+ is the most common phenotype of breast cancer constituting 66% of the tumors, followed by ER-/PR- (19%), ER+/PR- (12.5%), and ER-/PR+ (3.4%). Among these different tumors, ER-/PR- tumors are associated with the worst cancer-specific survival and are resistant to tamoxifen treatment [45]. Our results show that higher expression of PIPKIγ correlates to lower expression of ER and/or PR and correlates to lower patient survival rates. It is consistent with the fact that ER-/PR- tumors are associated with poor breast cancer prognosis. Although it is not clear if PIPKIγ could directly modulate ER or PR expression and if PIPKIγ could regulate ER or PR signaling, our results provide evidence that PIPKIγ correlates with ovarian hormone receptors status. It is worthy to demonstrate the possible role of PIPKIγ in regulating ovarian hormone pathways in breast cancer progression.

## Conclusions

The inverse correlation between strong PIPKIγ expression and overall patient survival is consistent with the finding that PIPKIγ is required for breast epithelial cell adherent junction assembly, growth factor stimulated migration, invasion, and proliferation. This study provides evidence of the pathological significance of PIPKIγ in breast cancer progression.

## Abbreviations

ARF6: ADP-ribosylation factor 6; BSA: bovine serum albumin; DMEM: Dulbecco's modified eagle medium; EGFR: epidermal growth factor receptor; ER: estrogen receptor; FBS: fetal bovine serum; HER: human epidermal growth factor receptor; PBS: phosphate-buffered saline; PI4, 5P_2_: phosphatidylinositol 4,5-bisphosphate; PIPKIγ: type I gamma phosphatidylinositol phosphate kinase; PR: progesterone receptor; shRNA: short-hairpin RNA.

## Competing interests

The authors declare that they have no competing interests.

## Authors' contributions

YS wrote the manuscript, performed the breast cancer migration and invasion assay. DT performed TMA and analyzed the statistical data and helped to draft the manuscript. KL provided the anti-PIPKIγ antibodies and helped to draft the manuscript. NT performed breast cancer proliferation assay. SL helped to perform TMA and analyze the statistical data. DH and RAA are the research group leaders, evaluated the data, edited and approved the final manuscript to be published. All authors read and approved the final manuscript.
